# Real‐world experience of nivolumab in the treatment of poor performance status patients with advanced non‐small cell lung cancer

**DOI:** 10.1002/cnr2.1487

**Published:** 2021-06-30

**Authors:** M. Nazim Abbas, Myron Klevansky, Bogda Koczwara, Amitesh Chandra Roy, Shawgi Sukumaran, Sina Vatandoust, Christos Stelios Karapetis

**Affiliations:** ^1^ Department of Medical Oncology, Flinders Centre for Innovation in Cancer Flinders Medical Centre Bedford Park South Australia Australia; ^2^ College of Medicine and Public Health Flinders University Adelaide Australia

**Keywords:** nivolumab, non‐small cell lung cancer, performance status, real‐world

## Abstract

**Background:**

Nivolumab improves disease control and survival in advanced NSCLC in patients with good performance status (PS), but there is limited data on its efficacy in patients with poor PS.

**Aim:**

Primary objective of the study was to evaluate the efficacy and safety of nivolumab and examine the influence of PS on outcomes.

**Methods and Results:**

Retrospective analysis of patients treated with single‐agent nivolumab for advanced NSCLC at a single institution was performed.

Sixty‐six patients treated with nivolumab were identified (33 male) with a median age of 68.5 years. Fifty‐six (85%) patients were current or former smokers and 17 (26%) had brain metastasis. All patients had received prior chemotherapy, 39 (59%) patients received one and 27 (41%) had ≥2 prior lines of therapy. Median overall survival (OS) was 7.1 months (95%CI 3.61–11.3) in the overall study population. OS of patients with PS ≥2 at the start of treatment was 3.04 months (95%CI 1.64–7.36) as compared to 10.23 months (95%CI 7.06–18.9) with PS ≤1. The overall response rate was 7% (four patients had a partial response), 23 (40%) patients had stable disease; the overall disease control rate (partial response and stable disease) was 47%. Twenty‐six (40%) patients had PS ≥2 at the start of treatment and 2 (8%) of these patients developed a partial response, 4 (15%) had stable disease; the overall disease control rate was 23%. Fourteen (58%) patients with PS ≥2 had disease progression at the time of first disease response evaluation. In the overall population, 20% of patients experienced grade ≥3 treatment‐related adverse events (TRAEs), most commonly pneumonitis, hepatitis, and colitis. Fourteen TRAEs led to treatment discontinuation, 9 (23%) adverse events (AEs) in patients with PS ≤1 and 5 (19%) with PS ≥2. Fourteen (21%) patients died within 30 days of the last nivolumab treatment.

**Conclusion:**

There was no significant difference in toxicity leading to treatment discontinuation between the poor and good PS groups, but survival was shorter with poorer PS. PS appears to be an important prognostic factor and remains a relevant discriminator in the selection of treatment with immunotherapy for lung cancer.

## 1 BACKGROUND

Nivolumab is a fully human IgG4‐blocking monoclonal antibody directed against programmed cell death‐1(PD‐1). Improvement in overall survival (OS) was demonstrated with nivolumab in two separate studies for patients previously treated with platinum‐based doublet chemotherapy with squamous (9.2 vs. 6 months; HR 0.59)^1^ and non‐squamous (12.2 vs. 9.4 months; HR 0.73)^2^ non‐small cell lung cancer. Three‐year pooled analysis of the two studies showed 3 year OS rates of 17% with nivolumab as compared to 8% with docetaxel.^3^ In Australia, nivolumab was initially available through a compassionate access program, and in 2016 it was approved by Pharmaceutical Benefits Scheme (PBS), the Australian national government drug funding program. Treatment efficacy of nivolumab in patients with advanced NSCLC has been demonstrated for patients with a good performance status (PS) (Eastern Cooperative Group (ECOG) ≤1).^4^ The efficacy of immunotherapies in the setting of poor PS has not been extensively evaluated. Patients with a PS of two accounts for approximately 30%–40% of patients diagnosed with advanced NSCLC in clinical practice.^5^ Patients with poor PS have historically been excluded or underrepresented in the clinical trials due to poor outcomes and there is a paucity of data to guide the optimal treatment strategy in this population.

Recently few prospective studies have explored the outcomes with the use of immunotherapy in advanced NSCLC patients with poor PS. Data from Phase 3B/4 Checkmate 153 study evaluating a subgroup of patients based on PS showed lower estimated 6 months OS in patients with PS 2 (41%) than PS 0–1 (65%), however, there was no significant difference in toxicities for poor PS patients compared to the overall study population.^6^ Similarly, results from the Phase II Checkmate 171 study showed that patients with PS 2 had lower median OS, 3 months and 6 months survival rates as compared to the overall study population.^7^ CHECKMATE 169 evaluated the advanced NSCLC patient outcomes that were treated with nivolumab and progressed after ≥1 prior line of prior therapy. This study included patients with age ≥ 70 years and PS 2 however patients with CNS metastasis were excluded. Preliminary results from the Canadian cohort of this study showed comparable safety outcomes and lower OS for patients with PS 2 as compared to the overall population.^8^

We suspected that the use of nivolumab in the ‘real‐world’ outside of the restrictive entry criteria of clinical trials, would include patients with poor PS, with both patients and oncologists enthusiastic about trying this novel treatment approach.

The objectives of the analysis were to explore the ‘real‐world’ application of nivolumab with the evaluation of efficacy, safety with analysis of treatment‐related toxicities, and effect of PS on outcomes particularly OS.

## 2 METHODS

A retrospective analysis was conducted for patients with advanced NSCLC treated with nivolumab between May 2015 and June 2017 at Flinders Medical Centre in Adelaide, Australia. Patients included in the study received at least one prior line of platinum‐based chemotherapy and patients with positive driver mutation received targeted therapy and platinum‐based chemotherapy before receiving nivolumab. The PS of patients was measured in accordance with the ECOG PS classification.^4^ ECOG PS at the time of starting nivolumab was captured through a review of the medical records. Data was also collected including age, gender, histology, EGFR and ALK mutational aberrations, stage at diagnosis, smoking status, presence of CNS metastasis, previous and subsequent treatments, grade 3/4 adverse events (AEs) related to nivolumab, mortality within 30 days of last nivolumab treatment, response to treatment assessed by the treating physician and survival outcomes. OS was calculated from the date of the first administration of nivolumab treatment until the last date of follow‐up or the date of death of the patient. Time to progression was calculated from the date of commencement of nivolumab to the date of disease progression or death. Duration of response was measured from the date that response was first observed to the date of progression or the date of death or last follow‐up in those without demonstrated progression.

## 3 RESULTS

**FIGURE 1:**
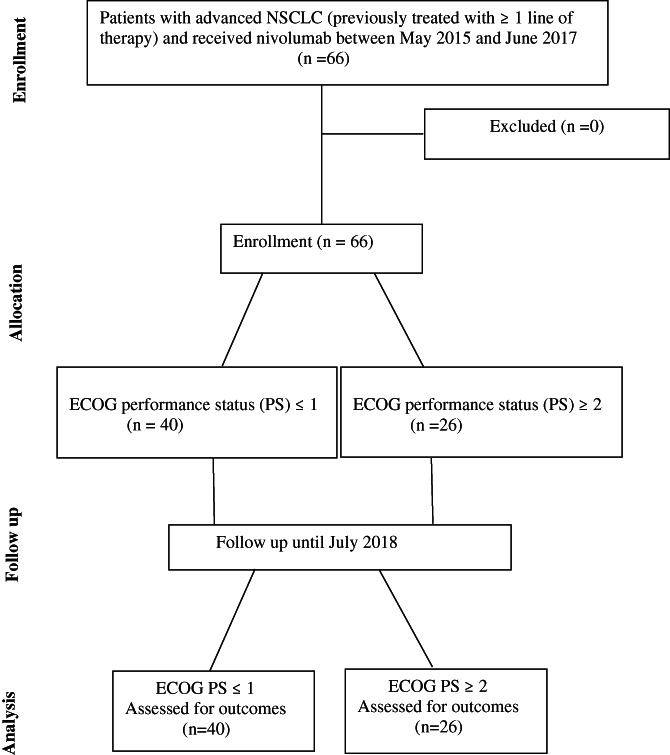
CONSORT diagram

Sixty‐six patients were treated with nivolumab for advanced NSCLC from May 2015 to June 2017 and data from all of these patients were included in this study (Figure 1).

Baseline demographics are shown in Table 1. The median age was 68.5 (range 43–85) years with an equal number of males and females (33 each) and 56 (85%) were former/current smokers. Patients with non‐squamous histology predominated; 38 (58%) adenocarcinoma, 23 (35%) squamous‐cell carcinoma, 2 (3%) adenosquamous, 2 (3%) large cell neuroendocrine carcinoma and 1 (2%) NOS (not otherwise specified). The majority of the patients had Stage IV cancer (*n* = 40; 61%) at the time of their initial lung cancer diagnosis. Twelve (18%) patients had KRAS mutation, two patients were EGFR mutation‐positive (3%), one patient had a BRAF mutation (2%) and no patient had lung cancer that was ALK mutation‐positive. ROS1 or PD‐L1 status was not routinely performed in our institute during the study period, however, PDL1 status for three patients was available (20%, 3%, and 0% PD‐L1 expression). Mutation analysis data were missing for three patients.

**TABLE 1:** Demographics

*N* = 66	
*Age*	Median 68.5 range (43–85)
*Gender*	
Male	33 (50%)
Female	33 (50%)
*ECOG PS (Pre‐nivolumab)*	
0	3 (4%)
1	37 (56%)
2	21 (32%)
3	5 (8%)
*ECOG PS (worse recorded during treatment)*	
0	0
1	19 (29%)
2	31 (47%)
3	16 (24%)
*Smoking status*	
Current smoker	18 (27%)
Former smokers	38 (58%)
Never smoked	10 (15%)
*Mutations*	
BRAF	1 (2%)
EGFR	2 (3%)
KRAS	12 (18%)
Nil	48 (73%)
Missing	3 (4%)
*CNS involvement*	
Brain metastasis	17 (26%)
No brain metastasis	49 (74%)
*Number of treatments*	
1 Prior line of therapy	39 (59%)
2 Prior lines of therapy	27 (41%)
*Type of prior systemic treatment*	
Platinum‐based chemotherapy	66 (100%)
Platinum‐based chemoradiotherapy	18 (27%)
Pemetrexed	18 (27%)
EGFR tyrosine kinase inhibitor	2 (3%)
*Histology*	
Adenocarcinoma	38 (58%)
Squamous cell carcinoma	23 (35%)
Adenosquamous	2 (3%)
Large cell neuroendocrine carcinoma	2 (3%)

Abbreviations: ECOG, Eastern Cooperative Group; PS, performance status.

PS ≥2 was recorded for 26 (40%) patients and 40 (60%) had PS ≤1. In the study population, 28 (42%) patients were ≥ 70 years old and the majority of patients had PS ≤1 (*n* = 18) and 10 (15%) patients had PS ≥2. Seventeen patients (26%) had CNS metastases in the overall population and the majority (*n* = 9) had PS ≥2 at the time of nivolumab commencement. All patients have previously received at least one platinum‐based chemotherapy and 27 (41%) patients received two or more prior systemic therapy lines (Table 1).

**FIGURE 2:**
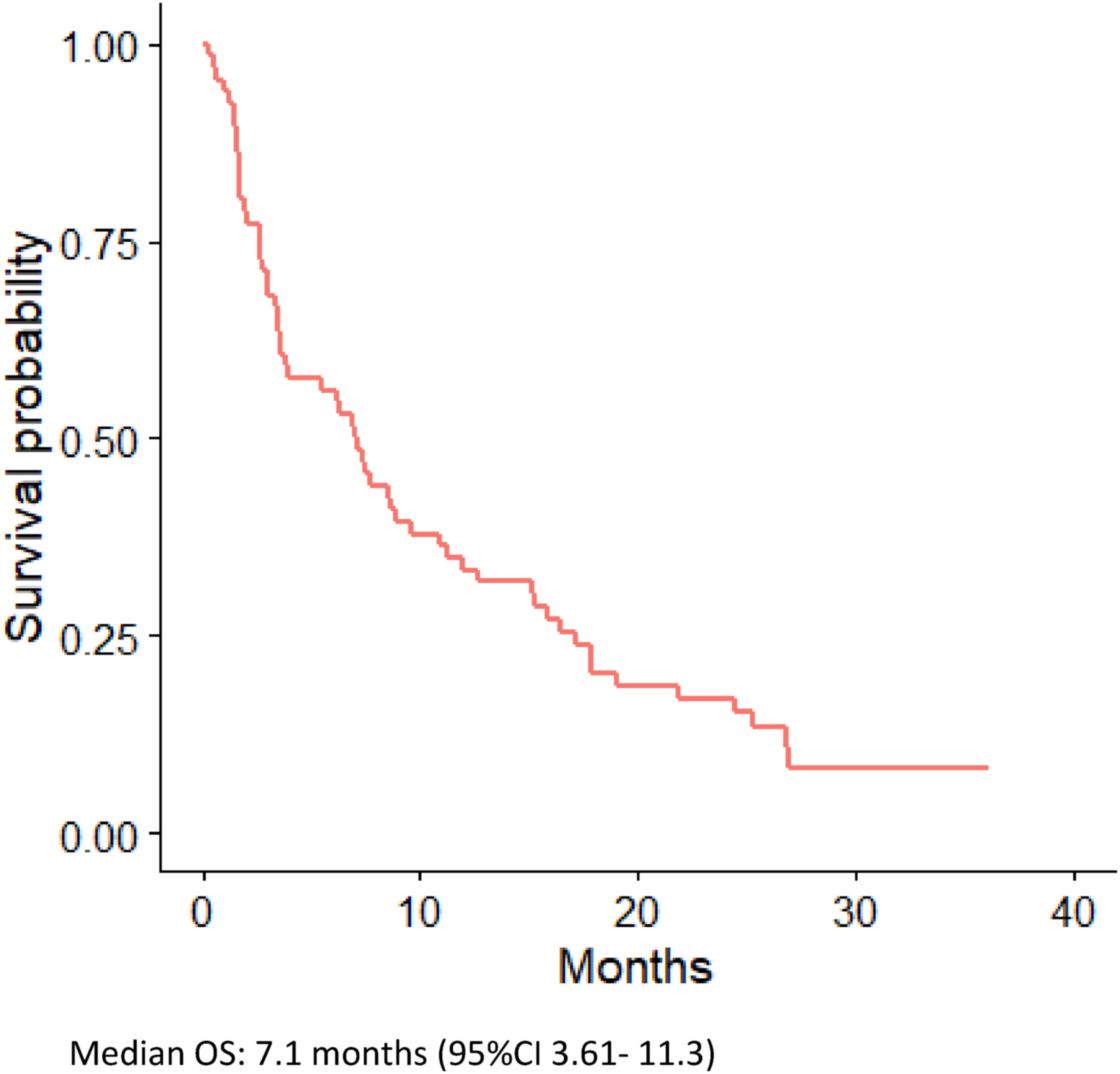
Overall survival (OS) – All patients

**FIGURE 3:**
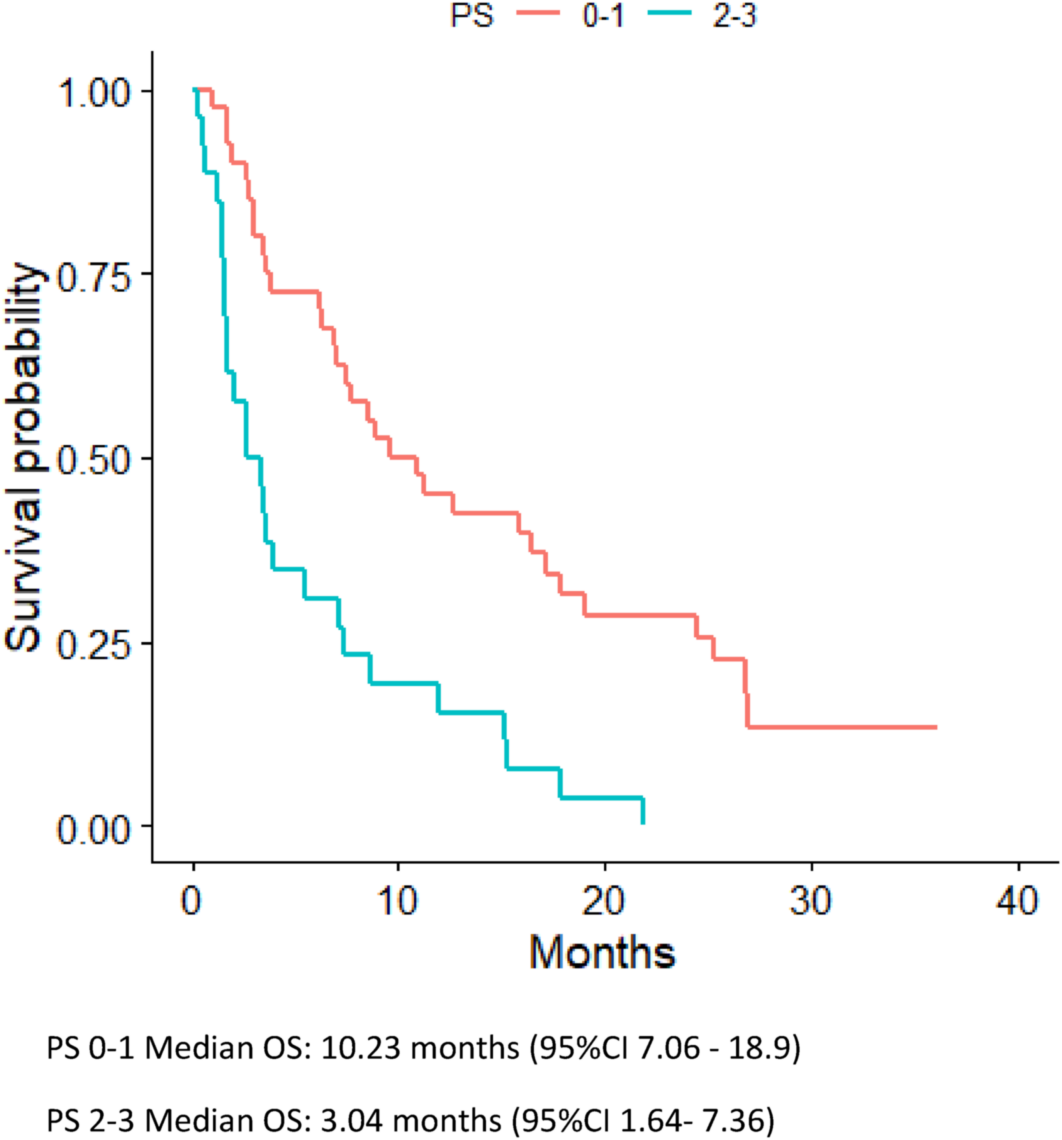
Overall survival (OS) based on performance status (PS)

All patients in the study received at least one dose of nivolumab. Median duration of treatment was 2.56 months (95%CI 1.9–3.2). Fourteen (21%) patients died within 30 days of last nivolumab treatment (eight patients had PS ≥2) and the cause of death was disease progression for 10 patients and two patients each died due to infection and treatment‐related pneumonitis respectively. Median OS of the whole included population was 7.1 months (95%CI 3.61–11.3) (Figure 2), 10.23 months (95%CI 7.06–18.9) for PS ≤1 and 3.04 months (95%CI 1.64–7.36) for PS ≥2 (Figure 3) (Table 2). Two patients (8%) with PS ≥2 at the start of nivolumab achieved a partial response and their survival was 12 and 7.4 months as compared to two patients (5%) with PS ≤1 group that survived 9.5 and 36.1 months. Two patients with ECOG PS ≥2 had a recorded improvement in PS while receiving treatment with nivolumab.

**TABLE 2:** Survival

Death within 30 days of last nivolumab dose	14 (21%)
Median duration of nivolumab therapy (months)	2.56 (95%CI 1.9–3.2)
Median duration of response (months)	
Median time to progression (months)	7.6 (95%CI 1.3–13.8)
2.2 (95%CI 1.4–7.1)	
Number of patients continuing nivolumab	2
Median OS (all patients)	7.1 months (95%CI 3.61–11.3)
Median OS PS < 1	10.23 months (95%CI 7.06–18.9)
Median OS PS ≥ 2	3.04 months (95%CI 1.64–7.36)

Abbreviations: OS, overall survival; PS, performance status.

Four (7%) patients had a partial response (PR) and 23 (40%) had stable disease (SD), making the disease control rate (PR + SD) 47%. No complete response was noted in the study population. Eight percent (two patients) of the patients with PS ≥2 had PR as compared to 5% (two patients) with PS ≤1 (Table 3). Median duration of response (DoR) was 7.6 months (PS ≤1; 10.1 months and PS ≥2; 5.5 months) and the median time to progression was 2.2 months. Treatment response to nivolumab could not be evaluated in five patients.

**TABLE 3:** Efficacy

*Best overall response*	*N* = 66
CR	0
PR	4 (7%)
SD	23 (40%)
PD	31 (53%)
Unable to evaluate	8 (12%)
*Best overall response ‐ PS ≤ 1*	*N* = 40
CR	0
PR	2 (5%)
SD	19 (48%)
PD	16 (40%)
Unable to evaluate	3 (7%)
*Best overall response ‐ PS ≥ 2*	*N* = 26
CR	0
PR	2 (8%)
SD	4 (15%)
PD	15 (58%)
Unable to evaluate	5 (19%)

Abbreviation: CR, complete response; PD, disease progression; PR, partial response; PS, performance status; SD, stable disease.

As shown in Table 4, in the overall study population grade three or higher TRAEs occurred in 14 (21%) patients leading to treatment discontinuation (9 (23%) AEs in patients with PS ≤1 and 5 (19%) with PS ≥2). Grade 3/4 AEs comprised of four patients with pneumonitis, three with hepatitis, two had colitis, and one each of hypophysitis, arthritis, and hypothyroidism. There was one death with pneumonitis that occurred in a patient with a PS of one.

**TABLE 4:** Adverse events

*Grade 3–4 TRAEs*	*N* = 12
Pneumonitis	4 (33%)
Hepatitis	3 (25%)
Colitis	2 (17%)
Hypophyisitis	1 (8%)
Arthritis	1 (8%)
Hypocortisolism	1 (8%)
*Grade 5 TRAEs*	
Pneumonitis	1
*TRAEs led to treatment discontinuation*	13
PS ≤ 1	8 (62%)
PS ≥ 2	5 (38%)

Abbreviations: PS, performance status; TRAEs, treatment‐related adverse events.

## 4 DISCUSSION

In this retrospective, single‐institution analysis, we assessed the real‐world experience of the application of nivolumab in advanced NSCLC and explored the influence on outcomes related to patients with poor PS. We found that 40% of patients had a poor PS before starting nivolumab and these patients had shorter OS as compared to patients with good PS. Although the study population was small there was no significant difference in response rates and toxicities experienced between the two PS groups.

In the pre‐immunotherapy era, PS was considered a strong predictor for treatment tolerability and response for the patients treated with chemotherapy.^9^, ^10^ Assessment of PS is subjective and can vary based on patient‐reported descriptions and the clinical assessment of the physician.^11^, ^12^ ECOG PS only takes into account the assessment of the functional ability of the patient. Factors like cancer‐related symptoms, comorbidities, cancer burden, and potentially reversible causes temporarily affecting the PS are not included in the assessment. All patients with PS ≥2 are not necessarily comparable and response to treatment may be different between these individuals. Currently, there are no clear guidelines to help to choose the most suitable patients with poor PS to receive benefits with immunotherapy. Prospective data to support the use of immunotherapies in patients with poor PS is limited to a few studies.^6^, ^8^, ^13^ Although ESMO guidelines^14^ do support patients with PS 2 receiving immunotherapy at the treating physician's discretion, these recommendations are based on extrapolation from the immunotherapy studies that recruited patients with PS 0–1. Retrospective studies that explored the outcomes of patients with poor PS in the advanced NSCLC population receiving immunotherapy showed similar results to our study highlighting shorter survival for patients with PS ≥2. In an Israeli retrospective study, patient outcomes in terms of survival and toxicity were evaluated.^15^ Forty‐six percent of patients in the study had ECOG PS ≥2. Median OS in the overall population was 5.9 months and both univariate and multivariate analysis showed only ECOG PS had a significant correlation with OS. Patients with PS 2 had 3.5 months OS as compared to 9.5 months for PS0/1. In a Japanese real‐world retrospective study, 141/603 patients had poor PS. Multivariate analysis showed poor PS was an independent negative predictor of poor PFS.^16^ In another observational study evaluating the real‐world experience with nivolumab in pre‐treated NSCLC, patients with PS2 had OS of 3.4 months as compared to 11.79 months OS for patients with PS 1.^17^

Our series demonstrates that patients in real‐world practice, outside the clinical trials, receive immune therapy despite having a poor PS. We suggest that in the majority of the cases these decisions are driven by the enthusiastic approach of the treating physician or in some cases strong patient wishes to try the immunotherapy with anticipation that immunotherapy will be less toxic and better tolerated as compared to chemotherapy. When making decisions to treat patients having poor PS with immunotherapy the use of validated tools like comprehensive geriatric assessment and Charlson comorbidity index that incorporate the assessment of comorbidities and frailty of patients could be considered.^18^

In our study partial response was achieved by four patients in the study and median DoR was 7.6 months. Median DoR for PS ≥1 was better than for patients with PS ≥2. Despite small numbers, patients with PS ≥2 seem to indicate shorter survival and duration of response. In the Checkmate 171 study patients with PS 2 had a response rate of 2.6% as compared to 11% in the overall population.^7^ These results further highlight the importance of considering PS as an important and independent indicator for poor outcomes.

Poor PS and CNS metastasis are generally considered as worse prognostic factors leading to inadequate outcomes and patients with age ≥ 70 are frequently frail and under‐represented or considered ineligible in clinical trials. Two Italian EAPs evaluated patients treated with nivolumab after failure with ≥1 prior therapy and CNS metastasis related to advance squamous^19^ and non‐squamous^20^ NSCLC and showed comparable outcomes as compared to the overall population in both analyses. In our study 28 (42%) patients were ≥ 70 years and the majority had PS ≤1 (*n* = 18) and 10 patients had PS ≥2. Seventeen patients (26%) who had CNS metastases out of these majority (*n* = 9) had PS ≥2. In the real‐world experience management of elderly and frail patients as well as patients with CNS metastasis is challenging and although we did not evaluate the specific outcomes for these patients, our study population is indicative of these practical management issues. Further studies are required to specifically evaluate these subsets to help guide appropriate treatment with benefit.

One of the limitations of our study is the small population size from a single institution and due to this, we could not further evaluate the immunotherapy‐related outcomes in a specific subset of patients in addition to the potential impact of the use of antibiotics and steroids on the outcomes. Despite these limitations, the heterogeneity of the real‐world patient cohort and outcomes consistent with the existing data does add to the importance of considering PS as an important factor before starting the immunotherapy.

In conclusion, in this retrospective real word study, we found that patients with poor PS have shorter OS as compared to good PS, but there was no significant difference in tumor response rates or safety outcomes based on PS. Further studies are needed to define the optimal treatment approach for patients with poor PS.

## CONFLICT OF INTEREST

Christos Stelios Karapetis has served on the advisory board for BMS, ROCHE, and AstraZeneca.

The rest of the authors have no disclosures and declare to have no conflicts of interest concerning this manuscript.

## AUTHOR CONTRIBUTIONS

All authors had full access to the data in the study and take responsibility for the integrity of the data and the accuracy of the data analysis. *Conceptualization*, C.S.K.; *Methodology*, M.N.A., M.K., C.S.K.; *Formal Analysis*, M.N.A., C.S.K.; *Writing‐Original Draft*, M.N.A., C.S.K.; *Writing‐Review & Editing*, M.N.A., M.K., B.K., A.C.R., S.S., S.V., C.S.K.; *Supervision*, M.N.A., M.K., B.K., A.C.R., S.S., S.V., C.S.K.; *Data Curation*, M.N.A., M.K.

## ETHICAL STATEMENT

The study was performed as a quality assurance project and institutional ethical approval was not required.

## Data Availability

The data that support the findings of this study are available on request from the corresponding author. The data are not publicly available due to privacy or ethical restrictions.
